# The Relationship Between Fears of Compassion, Emotion Regulation Difficulties, and Emotional Eating in College Students: A Moderated Mediation Model

**DOI:** 10.3389/fpsyg.2021.780144

**Published:** 2021-12-30

**Authors:** Shuwei Zhang, Mingchun Guo, Jingyun Wang, Lihua Lin

**Affiliations:** ^1^School of Health, Fujian Medical University, Fuzhou, China; ^2^School of Psychology, Fujian Normal University, Fuzhou, China

**Keywords:** fears of compassion, emotional eating, emotion regulation, college students, moderated mediation model

## Abstract

Although previous research has found the impact of fears of compassion on eating disorders, the mechanism underlying the relationship between fears of compassion and emotional eating remains to be examined. This study aimed to examine the mediating role of emotion regulation difficulties in the relationships between fears of compassion and emotional eating in college students, as well as the gender difference in the mediation model. The Fears of Compassion Scale, Difficulties in Emotion Regulation Scale, and Dutch Eating Behavior Questionnaire were completed by 673 college students in Fujian Province, China. Structural Equation Modeling was primarily employed to analyze the data. The results showed that both fear of compassion for self and fear of compassion from others were positively associated with emotion regulation difficulties, which in turn were related to emotional eating for female college students. Emotion regulation difficulties played a significant mediating effect in the relationship between fears of compassion and emotional eating. Comparatively, for male college students, only fear of compassion for self was positively associated with emotion regulation difficulties, but emotion regulation difficulties were not related to emotion eating. Moreover, the mediating effect of emotion regulation difficulties was not significant in the relationship between fears of compassion and emotion eating for male college students. The findings suggest that it is important to improve individuals’ fears of compassion to reduce emotional eating, particularly for female college students.

## Introduction

Eating disorders are a group of chronic mental disorders including anorexia nervosa, bulimia nervosa and binge eating, which are characterized by severe disturbances in eating behaviors and related thoughts and emotions and negatively affect individuals’ physical and mental health. Compared with the prevalence rates of eating disorders in the United States, which ranges from 0.28 to 0.85% across different studies (Udo and Grilo, 2018), the prevalence rate of eating disorders in China is less than 0.1% (Huang et al., 2019). However, subclinical conditions such as emotional eating are quite common among Chinese people, affecting 55.4% of girls and 48.4% of boys in Chinese college students (Liu et al., 2020). Considering the large population in China, it implies that a large number of Chinese young people are experiencing the negative impacts of eating problems. Therefore, it is important to understand the mechanism of emotional eating to inform psychological interventions for improving abnormal eating behaviors in Chinese youth, so as to reduce the prevalence rate of the subclinical conditions and prevent them from turning into clinical conditions.

### Emotional Eating and Emotion Regulation Difficulties

Emotional eating refers to eating that is triggered by emotions as opposed to a physiological need for food (Arnow et al., 1995). Emotional eating theory posited that negative emotions increase the motivation to eat and subsequently lead to eating behaviors, which in turn reduces the intensity of negative emotions (Macht and Simons, 2011). Numerous empirical studies have demonstrated that negative emotions stimulate more food consumption (Macht et al., 2004; Frayn and Knäuper, 2018), and individuals experience a decrease in negative emotions when they eat food such as sandwiches (Haedt-Matt et al., 2014; Litwin et al., 2017), providing support for emotional eating theory (Macht and Simons, 2011).

When people have negative emotions, they tend to use a range of different strategies to regulate their emotions, such as cognitive reappraisal, acceptance, distraction, and social sharing (Aldao and Dixon-Gordon, 2014). However, some individuals have emotion regulation difficulties, including a lack of awareness, non-acceptance of emotions, and using ineffective regulation strategies (Gratz and Roemer, 2004). These people are more prone to experience longer and/or escalated negative emotions, compared with their counterparts without emotion regulation difficulties. According to emotional eating theory (Macht and Simons, 2011), they are likely to have more emotional eating behaviors. In fact, emotional eating is considered to be a maladaptive emotion regulation strategy by which individuals are temporarily relieved of negative emotions through eating a large amount of food, but it also brings individuals higher risks of depression (Clum et al., 2014), eating disorders (Masheb and Grilo, 2006), and obesity (Frayn and Knäuper, 2018). Indeed, Extensive research has shown that emotion regulation difficulties are significantly associated with emotional eating (Larsen et al., 2006; Crockett et al., 2015; Braden et al., 2020), implying further that emotion regulation difficulties may bring about more emotional eating.

### Compassion and Fears of Compassion

Compassion has received much attention from researchers in recent years, which refers to “a sensitivity to suffering in self and others with a commitment to try to alleviate and prevent it” (Gilbert, 2014). Compassion is considered as based on evolved mammalian caregiving and care-receiving motivations (Gilbert, 2015; Gilbert et al., 2019). It can occur in three directions, including compassion for others, compassion from others and compassion for oneself (Neff, 2003; Gilbert, 2009). Growing evidence has shown that helping individuals develop compassion for themselves and for others through compassion-based interventions has a positive impact on their physiological and mental health (Jain et al., 2007; Neff and Germer, 2013; Jazaieri et al., 2014; Di Bello et al., 2020; Kim et al., 2020).

However, in his clinical practice, Gilbert found that some individuals with high levels of self-criticism and shame had negative reactions such as fear and avoidance when confronted with compassion, which is referred to as fears of compassion. Fears of compassion can also occur in three directions, including fears of (a) compassion toward others, (b) receiving compassion from others, or (c) compassion for oneself (Gilbert et al., 2011). Increasing evidence has suggested that fears of compassion are associated with a range of mental disorders, such as alexithymia, phobia, eating disorder, obsessive-compulsive disorder, and personality disorder (Kelly et al., 2013; Naismith et al., 2019; Merritt and Purdon, 2020), and with high levels of psychopathological symptoms such as self-criticism, self-coldness, shame, depression, and anxiety (Gilbert et al., 2011, 2012; Dentale et al., 2017; Kirby et al., 2019).

Some research has demonstrated that individuals with a low level of self-compassion and high levels of fears of compassion (Kelly et al., 2013) are likely to have eating disorders. Moreover, a version of Compassion-focused therapy (CFT) designed for eating disorders (CFT-E) has been shown to effectively help patients develop compassion and improve symptoms of eating disorders (Goss and Allan, 2010, 2014). However, more research is needed to examine the relationship between fears of compassion and emotional eating and the process by which fears of compassion relate to emotional eating.

### Fears of Compassion and Emotion Regulation Difficulties

Gilbert (2009) has distinguished three types of evolved emotion regulation systems, including the threat system, the drive system, and the safeness system. The threat system is sensitive to internal and external threats, helping us to protect from danger. When individuals perceive some threat, this system is activated and individuals experience emotions such as fear, anxiety, and anger, preparing them to escape or fight with the threat. The drive system is sensitive to cues of resources, social status and reward, which helps us to initiate and maintain behaviors to survive and develop in the society. When this system is activated, individuals experience high-arousal emotions such as desire, excitement, and pride. Comparatively, the safeness system is sensitive to cues of care, kindness and affiliation, and individuals experience low-arousal emotions such as contentment, calmness, and peace when it is activated (Gilbert, 2009). Although the emotions individuals experience following the activation of the threat system can help them to enact self-protection strategies (e.g., fight or flight), individuals with over-activated threat system are prone to emotional problems such as anxiety and depression (Gilbert, 2009). In contrast, well-developed safeness system can help individuals to regulate negative emotions arising from the activation of the threat system by receiving compassion from others and/or being self-compassionate (Gilbert, 2009; Kirby, 2017).

Self-compassion and accepting compassion from others can activate the safeness system and bring individuals feelings of safeness and warmth (Kelly and Dupasquier, 2016), thus it can be seen as a powerful, adaptive emotion regulation ability. In fact, a systematic review has shown that self-compassion is significantly associated with individuals’ adaptive emotion regulation, which subsequently contributed to their mental health (Inwood and Ferrari, 2018). On the contrary, fears of compassion are rooted in negative early experiences and shame traumatic memories of interactions with caregivers (Gilbert et al., 2011; Kirby et al., 2019), making the safeness system underdeveloped and the threat system over-sensitive to the environment, so that the safeness system are unable to down-regulate the threat system and associated negative emotions (Gilbert, 2009). For example, an individual who experienced parental abuse might have over-sensitive threat system and be sensitive to other people’s kindness. They tend to see kindness as a threat and thus react with fear and resistance to compassion from others. Similarly, the individual would also have underdeveloped safeness system and hard to have compassion for themselves (Kelly and Dupasquier, 2016).

Since fears of compassion are associated with underdeveloped safeness system and over-sensitive threat system, individuals with fears of compassion are likely to have emotional regulation difficulties. Indeed, abundant literature has demonstrated that fear of compassion for self and fear of compassion from others are associated with various forms of emotional dysfunction (Gilbert et al., 2011; Kirby et al., 2019). Further, we believe that these individuals would tend to have more emotional eating because of their emotion regulation difficulties. Nevertheless, this hypothesis has yet to be examined.

### The Present Study

Examining the mechanism of emotional eating from the perspective of fears of compassion can provide a new insight into emotional eating, and help psychologists and practitioners use compassion-based interventions such as CFT to improve emotional eating. Therefore, this study aimed to examine the relationship between fears of compassion and emotional eating, as well as the mediating role of emotion regulation difficulties in the relationship. We hypothesized that fears of compassion would be positively associated with emotional regulation difficulties, which would in turn be positively related to emotional eating. Considering that college female students have more emotional eating than male students (Liu et al., 2020), it is possible that emotion regulation difficulties would be associated with emotional eating only for female students but not for male students. Therefore, the present study also tested the gender difference in the mediation model.

## Method

### Participants

Participants were 740 college students from four universities in Fujian Province, China. Sixty-seven students were removed from data analysis, because the participants (1) selected the “disagree to participate” option on the informed consent form; (2) completed questionnaires with no response variance (e.g., ticked one choice for the entire questionnaire); or (3) did not fill out questionnaires, giving a valid sample of *N* = 673 students including 223 male (33.1%) and 450 female (66.9%), with *M*age = 20.67, *SD* = 1.19. 28.4% of them are only-child, 71.6% of them have siblings. 29.6% of participants were from urban cities, and 70.4% of them were from town areas. 87.9% of participants were from intact original families, whereas12.1% were from divorce or single-parent families.

### Procedure

All procedures of this study were approved by the Academic Ethics Committee of Fujian Medical University. In this study, we delivered online questionnaires to college students in four universities in Fujian Province *via* a QR code, which was presented in a PowerPoint slide to students in classrooms. After participants scanned the QR code using mobile phone, they would read an informed consent form, and then they filled in the questionnaires in a quiet classroom. Trained data collectors explained how to complete the questionnaires to students, and they could decide not to participate in the study or withdraw from the study at any time.

### Measures

#### Demographic Information

A few items were used to collect demographic information including gender, age, number of siblings, areas where students come from and their family status.

#### Fears of Compassion Scale

The Fears of Compassion Scale (FCS) is a self-report measure composed of three subscales (Gilbert et al., 2011): (1) Fear of compassion for self (FCFS) with 15 items (e.g., “I worry that if I start to develop compassion for myself I will become dependent on it”); (2) Fear of compassion from others (FCFRO) with 13 items (e.g., “If I think someone is being kind and caring toward me, I ‘put up a barrier”’); (3) Fear of compassion for others (FCFO) with 10 items (e.g., “I worry that if I am compassionate, vulnerable people can be drawn to me and drain my emotional resources”). Participants were asked to rate on a 5-point Likert scale ranging from 0 (“Don’t agree at all”) to 4 (“Completely agree”). The scale has been validated in China and obtained a three-factor structure with 31 items in a sample of Chinese college students (Guo et al., 2021). Because FCFS and FCFRO have been shown to have a greater impact on individual psychological functioning in previous studies (Kirby et al., 2019), only these two subscales were used in this study, which obtained Cronbach’s alphas 0.95 and 0.91, respectively.

#### Difficulties in Emotion Regulation Scale

The Difficulties in Emotion Regulation Scale (DERS) is a 36-item scale used to assess six components of emotion regulation difficulties (Gratz and Roemer, 2004; Bardeen et al., 2012), including: (1) impulsiveness (e.g., “When I’m upset, I have difficulty controlling my behaviors”), (2) non-acceptance of emotions (e.g., “When I’m upset, I become angry at myself for feeling that way”), (3) difficulty engaging in goal-directed behavior in the presence of negative emotions (e.g., “When I’m upset, I can still get things done”), (4) limited emotion regulation strategies (e.g., “When I’m upset, I know that I can find a way to eventually feel better”), (5) lack of awareness of emotions (e.g., “I pay attention to how I feel”), (6) lack of emotional clarity (e.g., “I know exactly how I am feeling”). Items were rated on 5-point Likert scale ranging from 1 (almost never) to 5 (almost always). The scale obtained good psychometric properties in a sample of Chinese sample (Li et al., 2018). The above six dimensions obtained Cronbach’s alphas 0.72, 0.87, 0.77, 0.81, 0.60, and 0.75 in the present study. Because lack of awareness of emotions had very low correlation with other dimensions, and the Confirmatory Factor Analysis (CFA) showed a factor loading of less than 0.3 for this dimension, hence only the remaining five dimensions were used for further analysis in this study.

#### The Dutch Eating Behavior Questionnaire

The Dutch Eating Behavior Questionnaire (DEBQ) is a 33-item measure comprised of three subscales assessing emotional, external, and restrained eating (Van Strien et al., 1986). The 13-item Emotional Eating Subscale is commonly used to measure emotional eating (e.g., “Do you have the desire to eat when you are irritated?”). Participants were asked to rate on a 5-point Likert scale ranging from 0 (“Never”) to 4 (“Always”). The DEBQ has been translated into Chinese and validated in China with a Chinese sample (Wu et al., 2017). It should be noted that the entire DEBQ questionnaire was administered to participants, but only the emotional eating subscale was used for data analysis. The Cronbach’s alpha of the subscale was 0.96.

### Data Analysis

We used SPSS v.23.0 and Mplus v. 8.3 to analyze the data. Descriptive statistics and correlation analysis were conducted using SPSS v. 23.0. In Mplus v. 8.3, CFA was conducted to test the measurement model of the four latent constructs including fear of compassion for self, fear of compassion from others, emotion regulation difficulties and emotional eating. After that, Structural Equation Modeling (SEM) was employed to estimate the hypothesized mediation model. Model fit was deemed acceptable using the following indices and criteria: the comparative fit index (Bentler, 1990) and the Tucker–Lewis index (Bentler, 1990) > 0.90 (Hu et al., 1992; Fan et al., 1999; Marsh et al., 2004), the root mean square error of approximation (Steiger, 1990) and standardized root mean square residual (Joreskog et al., 1979) < 0.08 (Hu et al., 1992; Browne and Draper, 2006).

To achieve satisfactory model fit and reduce model complexity, item parcels were created and used as indicators of latent variables (Little et al., 2002). For emotion regulation difficulties, we used average item scores of each dimension as indicators of the latent construct based on the internal-consistency approach (Kishton and Widaman, 1994). Three parcels were created for fears of compassion and emotional eating using the item-to-construct balance approach (Little et al., 2002). The mediation effects were evaluated using the bias-corrected percentile bootstrap method with 1,000 resamples (MacKinnon, 2011). If the 95% CI (bias-corrected confidence intervals) of the indirect effects in the mediation model did not include zero, we considered the indirect effects to be statistically significant (MacKinnon, 2011). In addition, Multiple-group SEM with Wald test was used to examine gender differences in the mediation model (Wang and Wang, 2019).

## Results

### Preliminary Analyses

Means, standard deviations and correlations of the study variables are shown in Table 1. It can be seen that all study variables were positively correlated, forming the basis for the mediation analyses. Further, it can be seen that fear of compassion from others had higher correlation with the five dimensions of emotion regulation difficulties for female than for male. And female students’ emotion regulation difficulties also seem to be more correlated with their emotional eating than male students, providing a basis for testing the gender difference in the mediation model.

**TABLE 1:** Means, SD, and Correlations of the study variables (*N*_*female*_ = 450, *N*_*male*_ = 223).

	1	2	3	4	5	6	7	8	*M*	*SD*
(1) Fear of compassion for others	1	0.72**	0.34**	0.43**	0.28**	0.19**	0.22**	0.33**	2.58	0.71
(2) Fear of compassion for self	0.62**	1	0.40**	0.47**	0.27**	0.20**	0.20**	0.34**	2.65	0.82
(3) Non-acceptance of emotions	0.46**	0.39**	1	0.75**	0.33**	0.40**	0.40**	0.30**	2.52	0.88
(4) Limited emotion regulation strategies	0.50**	0.42**	0.74**	1	0.28**	0.58**	0.26**	0.25**	2.45	0.73
(5) Impulsiveness	0.26**	0.26**	0.40**	0.35**	1	0.27**	0.14*	0.75**	2.58	0.66
(6) Difficulty engaging in goal-directed behavior in the presence of negative emotions	0.28**	0.28**	0.53**	0.64**	0.28**	1	0.22**	0.16**	2.31	0.72
(7) Lack of emotional clarity	0.31**	0.32**	0.34**	0.42**	0.07	0.23**	1	0.17**	2.34	0.43
(8) Emotional eating	0.29**	0.27**	0.37**	0.35**	0.83**	0.18**	0.01*	1	2.15	0.85
*M*	2.47	2.46	2.59	2.40	2.89	2.31	2.36	2.51	-	-
*SD*	0.70	0.75	0.79	0.70	0.64	0.70	0.55	0.89	-	-

Correlations for female students are reported below the diagonal, and above for male students. * p < 0.05, ** p < 0.01, *** p < 0.001.

### The Moderated Mediation Model

**FIGURE 1:**
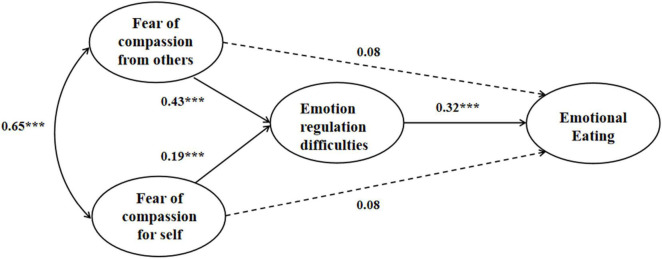
The mediation model of the study variables for female students; standardized coefficients are presented; * *p* < 0.05; ** *p* < 0.01; *** *p* < 0.001.

**FIGURE 2:**
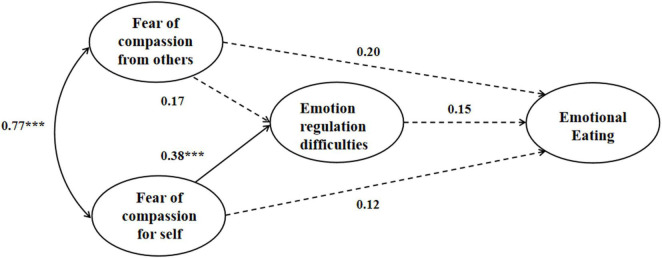
The mediation model of the study variables for male students; standardized coefficients are presented; * *p* < 0.05; ** *p* < 0.01; *** *p* < 0.001.

The hypothesized mediation models for both male and female showed adequate fit to the data. For the female mediation model (see Figure 1), χ^2^ = 147.39, *df* = 71, CFI = 0.99, TLI = 0.98, RMSEA = 0.05, SRMR = 0.04. As shown in Figure 1, for female college students, both fears of compassion were positively associated with emotion regulation difficulties, which were in turn related to emotional eating. Moreover, emotion regulation difficulties significantly mediated the relationship between fear of compassion for self and emotional eating [β = 0.062, *p* = 0.026, 95% CI (0.02, 0.13)], as well as the relationship between fear of compassion from others and emotional eating [β = 0.14, *p* < 0.001, 95% CI (0.08, 0.22)]. For the male mediation model, χ^2^ = 149.01, *df* = 71, CFI = 0.97, TLI = 0.96, RMSEA = 0.07, SRMR = 0.04. As shown in Figure 2, for male college students, only fear of compassion from others were positively associated with emotion regulation difficulties, which yet were not related to emotional eating. Moreover, emotion regulation difficulties did not significantly mediate either the relationship between fear of compassion for self and emotional eating [β = 0.057, *p* = 0.24, 95% CI (–0.01, 0.18)] or the relationship between fear of compassion from others and emotional eating [β = 0.03, *p* = 0.32, 95% CI (–0.01, 0.10)].

To test structural path coefficient invariance across the two mediation models, we first estimated a configural SEM model using male and female samples simultaneously, which served as a base model for model comparisons. The base model showed satisfactory fit to the data, χ^2^ = 337.71, *df* = 162, CFI = 0.98, TLI = 0.98, RMSEA = 0.06, SRMR = 0.05. After that, a series of multiple-group SEM with Wald tests were performed to examine the significance of differences in path coefficients and the mediating effects. The results showed that the two models were significantly different across male and female. Specifically, there was significant difference in the relationship between fear of compassion and emotion regulation difficulties (Wald test χ^2^ = 3.93, *df* = 1, *p* < 0.05), as well as the relationship between emotion regulation difficulties and emotional eating (Wald test χ^2^ = 4.32, *df* = 1, *p* < 0.05). Other path coefficients in the two models were not significantly different. Moreover, the mediating effect of emotion regulation difficulties was significantly different in the relationship between fear of compassion from others and emotion eating (Wald test χ2 = 4.98, *df* = 1, *p* < 0.05), and it was marginally significant in the relationship between fear of compassion for self and emotional eating (Wald test χ^2^ = 3.19, *df* = 1, *p* = 0.07).

## Discussion

In this study, a set of questionnaires were administered to college students in Fujian Province of China to examine the mediating role of emotion regulation difficulties in the relationship between fears of compassion and emotional eating, as well as the gender difference in the mediation model. The results showed that, for female students, both fears of compassion were positively associated with emotion regulation difficulties, which in turn were related to emotional eating. Moreover, emotion regulation difficulties significantly mediated the relationship between fears of compassion and emotional eating. In contrast, for male students, only fear of compassion for self was positively associated with emotion regulation difficulties, which yet was not related to emotional eating. The mediating effect of emotion regulation difficulties was not significant in the relationship between fears of compassion and emotional eating. It can be speculated that the gender difference in the mediating effects of emotion regulation difficulties are caused by the significant difference in the relationship between fear of compassion from others and emotion regulation difficulties, also between emotion regulation difficulties and emotional eating.

Regarding the gender difference in the relationship between fear of compassion from others and emotion regulation difficulties, we speculate that it can be explained by the following reasons. Compared with men, women have significantly larger social networks, and spend more time and energy to maintain their social networks and promote intimacy of social relationships (Antonucci and Akiyama, 1987; Haines et al., 2008). On one hand, women can receive more emotional support and compassion from their social networks. It can activate their safeness system and down-regulate their threat system, which helps to effectively cope with their negative emotions. However, on the other hand, women also need to devote more emotional resources to maintaining their social networks and may experience more stress of interpersonal conflict (Walen and Lachman, 2000; Turner et al., 2014). Therefore, when women have high levels of fear of compassion from others, they are less likely to receive emotional support and compassion from people in their social networks (i.e., their safeness system are more difficult to be activated in social relationships), and more likely to experience more stress of social interactions (i.e., their threat system are easier to be activated in social interactions), so that they are easier to have emotion regulation difficulties when encountering negative life events (Schneider et al., 2021).

Another interesting finding is that for male college students, emotion regulation difficulties were not associated with emotional eating, which is inconsistent with our hypothesis. According to Gilbert’s model of three emotion regulation systems, negative emotions such as anxiety and depression along with the activation of the threat system can be downregulated through the activation of the safeness system or the drive system (Gilbert, 2009). However, when an individual has high levels of fears of compassion and their safeness system is underdeveloped, they would have emotional regulation difficulties and may tend to rely more on the drive system to manage their negative emotions. Eating food is one way to activate the drive system to regulate their negative emotions. Nevertheless, the results of this study indicate that it is not the case for Chinese male college students. In contrast, two studies found significant relationship between emotion regulation difficulties and emotional eating for western college students (Kukk and Akkermann, 2017, 2020). Despite a lack of research on the relationship between emotion regulation difficulties and emotional eating for Chinese people, we speculate that Chinese male college students may use other ways to activate the drive system to manage negative emotions when they have emotion regulation difficulties. China is the world’s largest mobile games market, and the prevalence rate of gaming disorders is 17% (20.3% for males), much higher than 1.3–1.8% in Western countries (Liao et al., 2020; Xiang et al., 2020). Moreover, some research has shown that Chinese college male students play mobile games significantly more than female students (Long et al., 2018). Therefore, it is likely for Chinese college male students to play mobile games to gain virtual resources and achievements to regulate their negative emotions.

This study has several limitations. First, recruitment of participants was limited to Fujian Province, China; second, only self-reported measures were used in the study, which might lead to common method variance; third, this study employed a cross-sectional design, and thus could not judge the direction of the relationships between variables. Nevertheless, to our knowledge, this is the first study examining the relationship between fears of compassion and emotional eating in China, and the findings of this study have meaningful implications for clinical practice and future research.

Specifically, the results of this study showed that emotion regulation difficulties only played a significant mediating role in the relationship between fears of compassion and emotional eating for female but not for male college students. The findings suggest that practitioners can use compassion-based interventions such as CFT-E to reduce female college students’ fears of compassion, which can help them improve their emotion regulation difficulties and eventually reduce emotional eating behaviors. More research is needed to examine the relationship between emotion regulation difficulties and emotional eating in other samples of Chinese male college students and examine why emotional regulation difficulties were not related to emotional eating in this study. In terms of compassion-based interventions for Chinese male college students, the interventions may need more adaptations to effectively reduce their emotional eating. Without doubt, it also needs more research to guide the adaptation of the interventions and test the effectiveness of compassion-based interventions on eating behaviors for Chinese college students.

## Data Availability Statement

The original contributions presented in the study are included in the article/Supplementary Material, further inquiries can be directed to the corresponding author.

## Ethics Statement

The studies involving human participants were reviewed and approved by Academic Ethics Committee of Fujian Medical University. The patients/participants provided their written informed consent to participate in this study.

## Author Contributions

SZ collected the data and wrote the manuscript. MG designed the research and revised the manuscript. JW analyzed the data and wrote the manuscript with SZ. LL was involved in research design and collected the data. All authors listed have made a substantial, direct, and intellectual contribution to the work, and approved it for publication.

## Conflict of Interest

The authors declare that the research was conducted in the absence of any commercial or financial relationships that could be construed as a potential conflict of interest.

## Publisher’s Note

All claims expressed in this article are solely those of the authors and do not necessarily represent those of their affiliated organizations, or those of the publisher, the editors and the reviewers. Any product that may be evaluated in this article, or claim that may be made by its manufacturer, is not guaranteed or endorsed by the publisher.
